# Endoscopic surgery for spontaneous supratentorial intracerebral haemorrhage: A systematic review and meta-analysis

**DOI:** 10.3389/fneur.2022.1054106

**Published:** 2022-12-20

**Authors:** Tim J. Hallenberger, Raphael Guzman, Leo H. Bonati, Ladina Greuter, Jehuda Soleman

**Affiliations:** ^1^Department of Neurosurgery, University Hospital Basel, Basel, Switzerland; ^2^Faculty of Medicine, University of Basel, Basel, Switzerland; ^3^Division of Paediatric Neurosurgery, Children's University Hospital of Basel, Basel, Switzerland; ^4^Department of Neurology, University Hospital Basel, Basel, Switzerland

**Keywords:** endoscopy, intracerebral haemorrhage, neurosurgery, meta-analysis, outcome

## Abstract

**Introduction:**

Treatment for spontaneous supratentorial intracerebral haemorrhage (SSICH) is limited and consist of either best medical treatment (BMT) or surgical hematoma evacuation. Treatment methods and choice of surgical technique are debated, and so far, no clear advantage of endoscopic surgery (ES) over conventional craniotomy (CC) or BMT was shown. The aim of this systematic review and meta-analysis was to investigate the differences in outcome, morbidity, and mortality between ES and CC or BMT.

**Methods:**

We systematically searched Embase and PubMed databases for randomised controlled trials comparing ES to CC or BMT. The primary outcome was favourable functional outcome after 6 months. Secondary outcomes were morbidity and mortality rates and duration of surgery.

**Results:**

Seven articles were eligible for the outcome analysis with 312 subjects in the control (216 CC, 96 BMT) and 279 in the treatment group (ES). Compared to BMT, ES showed significantly improved favourable functional outcome (RR 1.93 [1.12; 3.33], *p* = 0.02) and mortality rates (RR 0.63 [0.44; 0.90], *p* = 0.01). No significant difference in favourable functional outcome and mortality was seen in ES compared to CC (RR 2.13 [0.01; 737], *p* = 0.35; RR 0.42 [0.17; 1.05], *p* = 0.06). ES showed significantly lower morbidity (RR 0.41 [0.29; 0.58], *p* < 0.01), and overall infection rates (RR 0.33 [0.20; 0.54], *p* < 0.01) compared to CC. Duration of surgery was significantly shorter for ES compared to CC (SMD −3.17 [−4.35; −2.00], *p* < 0.01).

**Conclusion:**

ES showed significantly improved favourable functional outcome and mortality rates compared to BMT while showing reduced length of surgery and lower complication rates compared to CC. Therefore, ES appears a promising approach for treatment of SSICH justifying further prospective trials.

**Systematic review registration:**

PROSPERO, identifier: CRD42020181018.

## 1. Introduction

Spontaneous supratentorial intracerebral haemorrhage (SSICH) accounts for approximately 9–27% of all strokes, affecting more than 5 million people worldwide annually (1). In total of 60–70% are caused by hypertension and 5–20% are related to amyloid angiopathy and other non-structural reasons (2, 3). It is most commonly located in the basal ganglia but can also be of lobar origin (4, 5). Clinical outcome is often poor and known to be influenced by the primary hematoma volume, hematoma expansion, neurotoxic metabolites, as well as oedema (4). SSICH is associated with a 30-day mortality rate of up to 40% (6–9). Current treatment recommendations can be divided into best medical treatment (BMT), which is a combination of early SSICH diagnosis, strict blood pressure control, reversal of anticoagulation and in-patient management in dedicated stroke units or intensive care units, or into surgical evacuation of the hematoma (10, 11). The mainstay of surgical treatment is a reduction of the hematoma volume resulting in a reduction of the mass effect and prevention of secondary neurotoxic injury caused by haemoglobin breakdown metabolites (4, 12). Surgical hematoma evacuation can be achieved through conventional craniotomy (CC) or minimal invasive surgery (MIS), including hematoma reduction through catheter-based infusion of rtPA and endoscopic surgery (ES) (10, 11, 13). Decompressive craniectomy might reduce mortality in patients suffering mass effect due to severe SSICH by simply reducing intracranial pressure without evacuation of the clot (10). Despite several large randomised controlled trials (RCTs) comparing various surgical techniques to BMT, current treatment recommendations and guidelines are lacking consensus on whether, and if so, which surgical treatment should be applied in SSICH (10, 13–17). Over the years, several different systematic reviews with meta-analyses were carried out comparing various MIS techniques to CC or BMT, most recently by Scaggiante et al. (18), Yao et al. (19), Nam et al. (20), Zhao et al. (21), Guo et al. (22), Li et al. (23), Sondag et al. (24), and Hou et al. (25). Despite also reporting ES as main surgical technique or as a technique in a subgroup analysis, all previous works either mixed ES with other MIS techniques as the experimental group or compared ES to both BMT and CC as a combined comparator, effectively creating a mixture of different treatments in the control group, possibly limiting the generalizability of the results (18–24). The aim of this systematic review and meta-analysis was to investigate endoscopic hematoma evacuation in comparison to either CC and BMT separately regarding functional clinical outcome, morbidity, and mortality.

## 2. Material and methods

### 2.1. Search strategy, data analysis, and data extraction

We used a search string with the keywords “spontaneous intracerebral haemorrhage” and “endoscopic surgery” in both PubMed and Embase databases including studies published until the 1^st^ of June 2020 (Supplementary Figure 1). After removal of duplicates, titles and abstracts of remaining articles were reviewed by two of the authors independently (TJH and LG). In a second step, full text publications of selected abstracts were independently reviewed by the same authors, based on which a final list of eligible studies was compiled. The reference lists of the final articles were searched manually for further eligible studies. In case of disagreement on the inclusion of a study a third researcher (JS) took a final decision. Data were extracted from the included studies by two researchers (TJH, LG) and compiled to a final data set for analysis.

### 2.2. Inclusion and exclusion criteria and outcome measures

We included RCTs fulfilling the following criteria:

1) Inclusion of spontaneous supratentorial ICH confirmed by imaging.
2) Comparison of ES to either BMT or CC.
3) Inclusion of patients >18 years of age.
4) Written in English.

We excluded studies according to the following criteria:

1) Inclusion of patients with a secondary hematoma due to a tumour, vascular lesion or malformation or traumatic causes.
2) Description of other forms of MIS than ES (e.g., stereotactic aspiration or catheter lysis).
3) Not an RCT.

The primary outcome measure was favourable functional outcome at 6 months. Favourable functional outcome was defined as a modified Rankin Scale (mRS) of 0–3 points, a Barthel Index (BI) of ≥70 or an Activity of Daily Living (ADL) score of 1–3, respectively and a Glasgow Outcome Scale (GOS) of 4–5 Points. Due to inconsistency of the reported outcome measures among the studies, we chose to use the four most frequently reported scores to assess favourable functional outcome. Thresholds for the BI and ADL were chosen in accordance with the literature and the current consensus (26–28). For the GOS, scores 4 and 5 were chosen as they are quite similar to mRS 1–3 (29).

Secondary outcomes were mortality, morbidity, postoperative residual hematoma volume (defined as the hematoma volume after intervention in millilitres), hematoma evacuation rate (defined as the difference of hematoma volume before and after surgery calculated as percentage), and the duration of surgery (in minutes). Morbidity consisted of re-bleeding, seizure, revision surgery, and infection (including pulmonary infection, surgical site infection (SSI), and central nervous system (CNS) infections). We compared ES to CC and BMT individually. The pooled outcome analysis for hematoma evacuation rate, postoperative hematoma volume and the temporal evolution of mortality rates was initially not planned in the protocol but was added as exploratory analyses.

### 2.3. Quality assessment

We used the revised Risk of Bias (RoB 2) tool to evaluate the included studies (30). The RoB 2 tool is the recommended tool to assess risk of bias in RCTs by the Cochrane Collaboration and covers all aspect of trial design, conduct and reporting (30). Quality assessment was carried out independently by two authors (TJH and LG) and compared thereafter. This review was conducted in accordance with the Preferred Reporting Items for Systematic Reviews and Meta-Analyses (PRISMA) guidelines (31) and was registered at PROSPERO under the registration number CRD42020181018. No ethical approval was needed for the present study.

### 2.4. Statistical analysis

Relative risk ratio (RR) was used as an effect measurement for the pooled outcomes. Confidence intervals (CI) and *p*-values were calculated for each outcome. Results with *p*<*0.05* were considered statistically significant. To identify influential studies distorting the primary outcome, a leave-one-out analysis was performed. If low heterogeneity (*I*^2^<*50%*) was present, we used fixed-effects models, while otherwise the random-effects models were used. Forest plots were generated for each outcome parameter to evaluate publication bias. All statistical analyses were done using R statistical software (version 4.0.2, 2020, The R foundation, U.S.A) using the dmetar package (32).

## 3. Results

**Figure 1:**
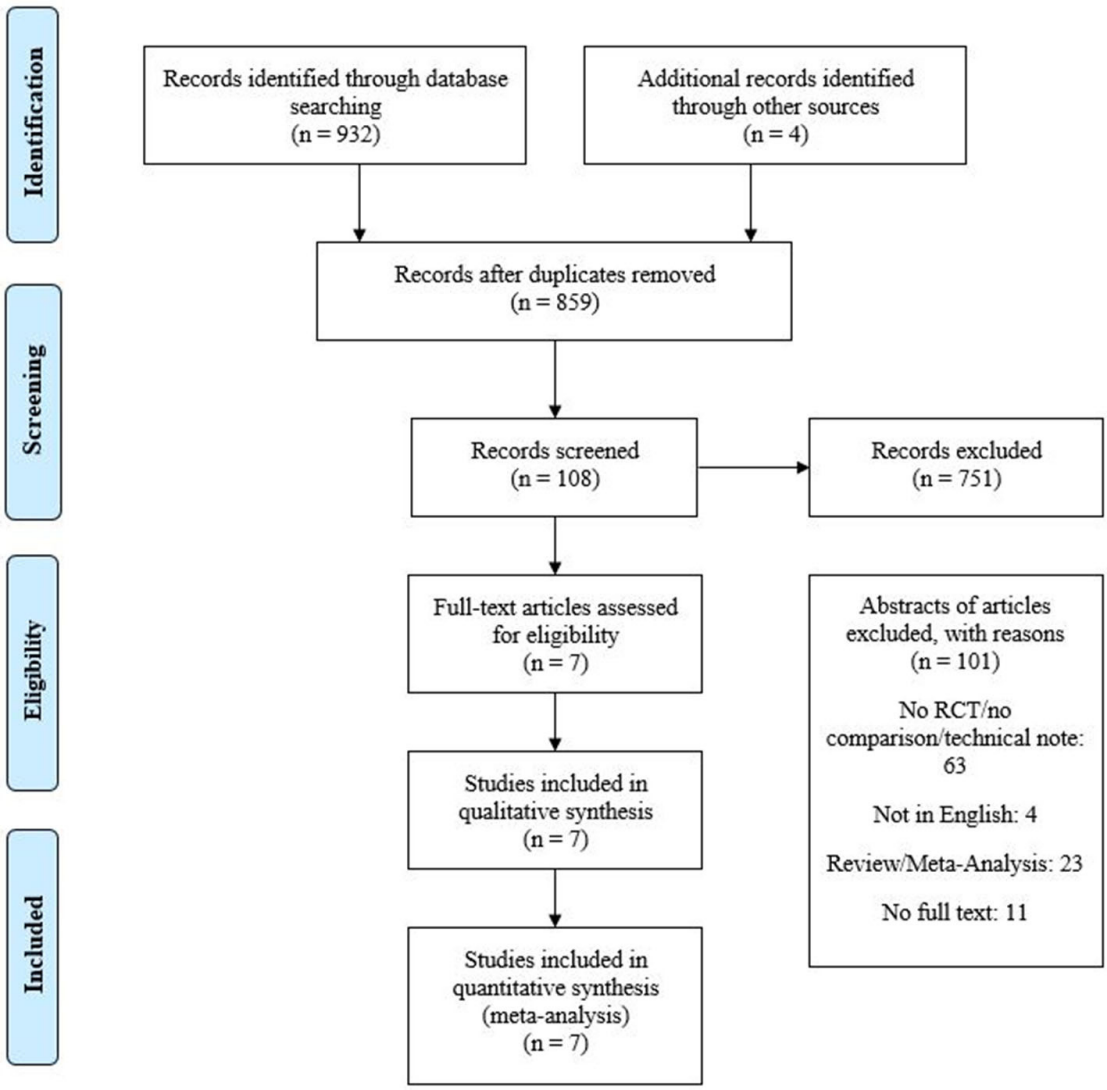
Flowchart of the study selection process according to the PRISMA guidelines.

A total of 932 articles were identified of which seven met the eligibility criteria, with a total of 591 patients, 312 subjects (52.8%) in the control group (216 subjects with CC and 96 subjects with BMT) and 279 subjects (47.2%) in the treatment group (ES) (Figure 1). ES was compared to BMT in three studies (33–35) and to CC in four studies, respectively (36–39) (Table 1). One RCT comparing ES to BMT to develop a modified intracerebral haemorrhage score and identify optimal cut-offs for surgical vs. conservative treatment in basal ganglia haemorrhage was reviewed and excluded since the primary outcome of the respective study did not compare the treatment modalities itself but rather when to apply them based on the score (40).

**Table 1:** Characteristics of the included studies.

**Study ID**	**C**	**Cases (T/C)**	**Mortality (T/N)**	**Mortality (C/N)**	**Location of SSICH**	**Time to treatment^†^**	**Primary outcome**	**Follow-up (months)**
Auer et al. (33)	BMT	50/50	21/50	35/50	Subcortical, putaminal, thalamic	Within 48 h	Mortality	6
Cho et al. (36)	CC	30/30	0/30	4/30	Basal ganglia	Within 24 h	Safety, outcome, cost effectiveness	6
Miller et al. (34)	BMT	6/4	1/6	2/4	Subcortical	Within 24 h	Safety, outcome, cost effectiveness	3
Zhang et al. (37)	CC	21/30	0/21	3/30	Basal ganglia	Within 24 h	Clinical outcome	6
Feng et al. (38)	CC	93/91	6/93	8/91	Subcortical, basal ganglia, internal capsule	Not specified	Outcome, cost effectiveness	6
Vespa et al. (35)	BMT	14/42	2/14	4/42	Deep (not otherwise specified), lobar	Within 48 h^‡^	Safety, neurological outcome	12
Zhang et al. (39)	CC	65/65	Not reported	Not reported	Lobar, basal ganglia	Not specified	Outcome, safety	1

T, treatment group; C, control group; N, total number of patients allocated to group; CC, conventional craniotomy; BMT, best medical treatment.

^†^after symptom onset if not stated otherwise.

^‡^after initial computed tomography.

### 3.1. Favourable functional outcome

**Figure 2:**
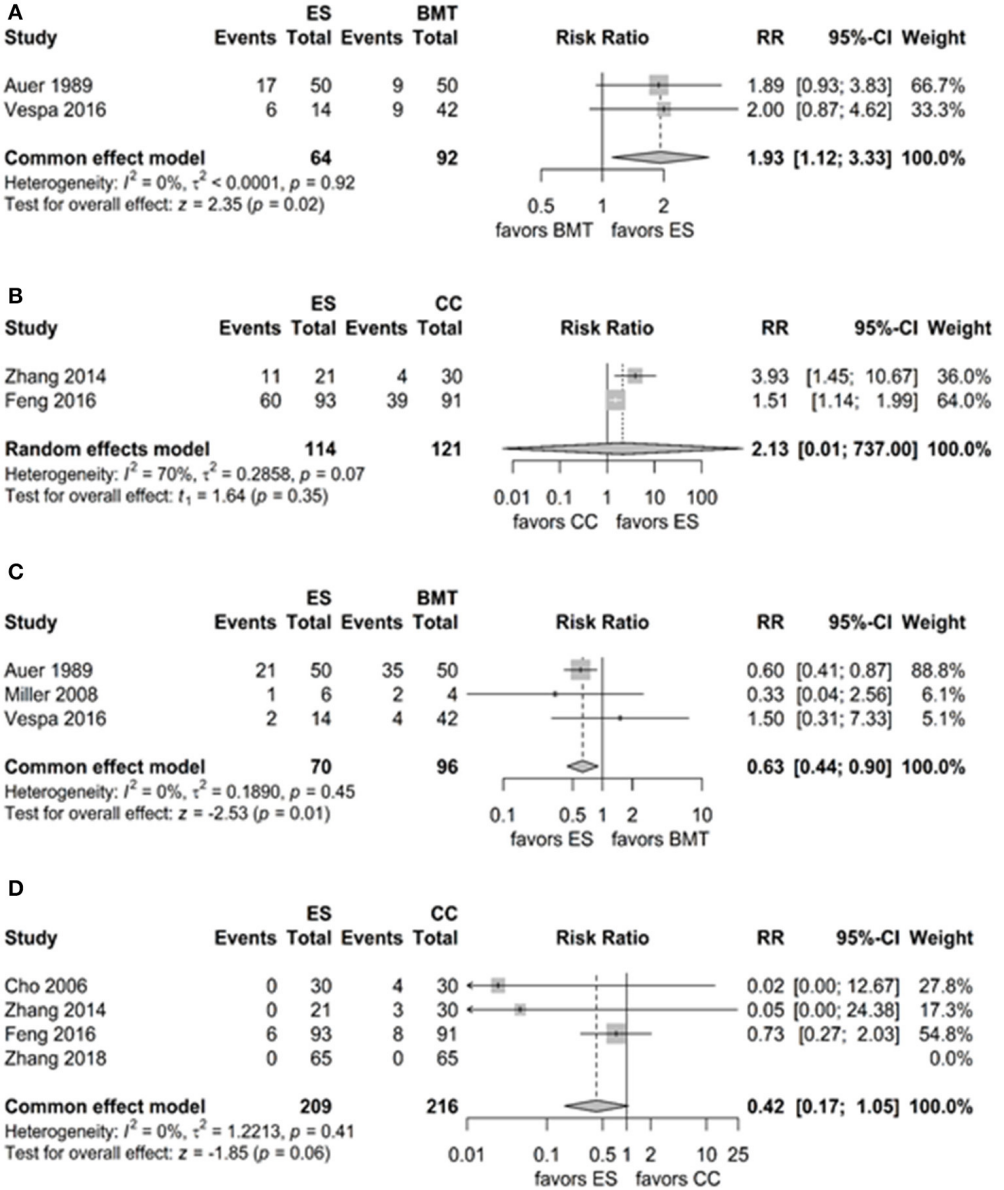
**(A)** Forest plots of favourable functional outcome endoscopic surgery (ES) vs. best medical treatment (BMT). **(B)** Favourable functional outcome ES vs. CC. **(C)** Mortality rates ES vs. BMT. **(D)** Mortality rates ES vs. CC.

Favourable functional outcome 6 months after treatment was reported in five of the seven included studies showing an overall rate of 38.3% (33, 35, 37, 38). ES showed a statistically significant higher rate of favourable functional outcome compared to BMT (*p* = 0.02, Table 2, Figure 2A). No significantly influential study was identified for ES vs. BMT after the “leave-one-out” method. Comparing ES to CC, a non-significant higher favourable functional outcome rate for ES with a moderate to high heterogeneity was observed (*p* = 0.35, Table 2, Figure 2B). After applying the “leave-one-out” method, Feng et al. (38) was identified as the influential study for ES vs. CC, however no pooled outcome analysis was possible as only one study remained for the analysis (37).

**Table 2:** Results of the meta-analysis for ES compared to BMT and CC.

**Results for endoscopic surgery compared to best medical treatment**						
**Outcome variable**	**Overall rates ES vs. BMT**	**No. studies included**	**RR [95% CI]**	**I** ^2^	**P-value for heterogeneity**	**P-value for overall effect**
Favourable functional outcome	35.9 vs. 19.6%	2	1.93 [1.12; 3.33]	0%	0.92	0.02
Mortality	34.3 vs. 42.7%	3	0.63 [0.44; 0.90]	0%	0.45	0.01
Overall complications	18.6 vs. 60.4%	3	0.43 [0.04; 4.36]	61%	0.08	0.26
Re-bleeding	8.6 vs. 21.9%	3	0.54 [0.02; 17.64]	72%	0.03	0.53
**Results for endoscopic surgery compared to conventional craniotomy**						
**Outcome variable**	**Overall rates ES vs. CC**	**No. studies included**	**RR [95% CI]**	**I** ^2^	**P-value for heterogeneity**	**P-value for overall effect**
Favourable functional outcome	62.3 vs. 35.35%	2	2.13 [0.01; 737.0]	70%	0.07	0.35
Mortality	2.9 vs. 6.9%	4	0.42 [0.17; 1.05]	0%	0.41	0.06
Overall complications	20.8 vs. 49.7%	3	0.41 [0.29; 0.58]	0%	0.62	< 0.01
Re-bleeding	3.9 vs. 10.0%	2	0.40 [0.08; 1.87]	0%	0.82	0.24
Overall infections	11.1 vs. 33.7%	3	0.33 [0.20; 0.54]	0%	0.76	< 0.01
Pneumonia	13.2 vs. 38.8%	2	0.33 [0.19; 0.55]	0%	0.44	< 0.01

ES, endoscopic surgery; BMT, best medical treatment; CC, conventional craniotomy; RR, relative risk; CI, confidence interval; I^2^, heterogeneity.

### 3.2. Mortality and morbidity

Mortality was reported in all of the included studies showing an overall rate of 21.7% (33–39). A significantly lower rate of mortality was observed for ES compared to BMT (*p* = 0.01, Table 2, Figure 2C). ES compared to CC showed a non-significantly lower mortality rate (*p* = 0.06, Table 2, Figure 2D). Mortality for ES was reported in four studies. Auer et al. reported a mortality rate of 42% (21/50), Miller et al. of 20% (1/6), Vespa et al. of 14% (2/14) and Feng et al. of 6% (6/93) for ES (33–35, 38). Cho et al., Zhang et al., and Zhang et al. reported no fatalities for ES (37, 39, 40). Mortality for CC was reported in three studies. Cho et al. reported a mortality rate of 13% (4/30), Zhang et al. of 10% (3/30) and Feng et al. of 9% (8/91) (37, 38, 40). Zhang et al. reported no fatalities (39). Mortality for BMT was reported in three studies. Auer et al. reported mortality rates of 70% (35/50), Miller et al. of 50% (2/4) and Vespa et al. of 10% (4/42) (33–35).

Morbidity was reported in six out of seven studies (33–38). Overall complication rate was 37.4% and significantly lower in ES than in CC (*p* < 0.01, Table 2, Supplementary Figure 2B). In contrast to BMT, ES showed a lower rate of overall complications but did not reach statistical significance (*p* = 0.26, Table 2, Supplementary Figure 2A).

Re-bleeding rate was reported in five studies (33–37). The overall re-bleeding rate was 11.1% and when ES was compared to CC or BMT no significant difference was observed (*p* = 0.24 and *p* = 0.53 respectively, Table 2, Supplementary Figures 3A, B).

The overall infection rate was 25.5% and was reported in four studies (35–38). Comparing ES to CC, a significantly lower rate of overall infections (*p* < 0.01, Table 2, Supplementary Figure 3C) and a lower rate of pneumonia (*p* < 0.01, Table 2, Supplementary Figure 3D) was observed. Regarding CNS infections and surgical site infections (SSI), no pooled outcome analysis was possible as only one study per control group reported these outcome measures (35–37).

Seizure rates were reported in two out of seven studies with a total rate of 26.0% (35, 38). However, no pooled outcome analysis was possible, because the two studies reporting seizure rates, each belonged to a different control group (35, 38).

### 3.3. Post-operative hematoma volume, hematoma evacuation rate, and duration and timing of surgery

Three of the included seven studies [one being compared to BMT (35)] analysed the postoperative hematoma volume [7.89 ml (±6.53) for ES vs. 15.02 ml (±10.06) for CC] while five studies [one being compared to BMT (34)] compared the hematoma evacuation rate [86.02% (±10.53) for ES vs. 77.6% (±4.79) for CC] after intervention (34–39). No difference in the hematoma evacuation rate nor the postoperative hematoma volume was found when comparing ES to CC with significant heterogeneity was observed (*p* = 0.07 and *p* = 0.44 respectively, Table 3, Supplementary Figures 4A, B).

**Table 3:** Results of the meta-analysis for ES compared to CC.

**Outcome variable**	**No. studies included**	**SMD** **[95% CI]**	**I^2^**	**P-value for heterogeneity**	**P-value for overall effect**
Postoperative hematoma volume	2	−2.12 [−24.26; 20.02]	99%	< 0.01	0.44
Hematoma evacuation rate	4	−1.08 [−2.32; 0.15]	92%	< 0.01	0.07
Duration of surgery	4	−3.17 [−4.35; −2.00]	93%	< 0.01	< 0.01

SMD, standardised mean difference; CI, confidence interval; I^2^, heterogeneity.

Duration of surgery was reported in four studies (36–39). ES had an average duration of surgery of 123.39 (±36.62) minutes in contrast to 273.78 (±85.55) minutes for CC. Pooled outcome analysis showed significantly shorter duration of surgery for ES vs. CC with significant heterogeneity (*p* < 0.01, Table 3, Supplementary Figure 4C).

Time to surgery was similar among the different studies (Table 1). All but two (38, 39) reported a treatment window, which was within 72 h (33–39). Three studies (34, 36, 37) defined a maximum treatment window of 24 h while two studies (33, 35) allowed a treatment window of 48 h. However, most patients in the latter two studies, were treated within 18–29 h after symptom onset (34, 35, 37). Only one of the included studies, reported “ultra-early” clot evacuation, in which patients were treated within the first 60–100 min after hospital admission (36). Due to the similarity of the described treatment windows in the included studies, no pooled outcome analysis was conducted. A regression analysis between time to treatment and mortality showed no significant association (*p* = 0.891) (34–36).

### 3.4. Quality assessment of included studies

**Figure 3:**
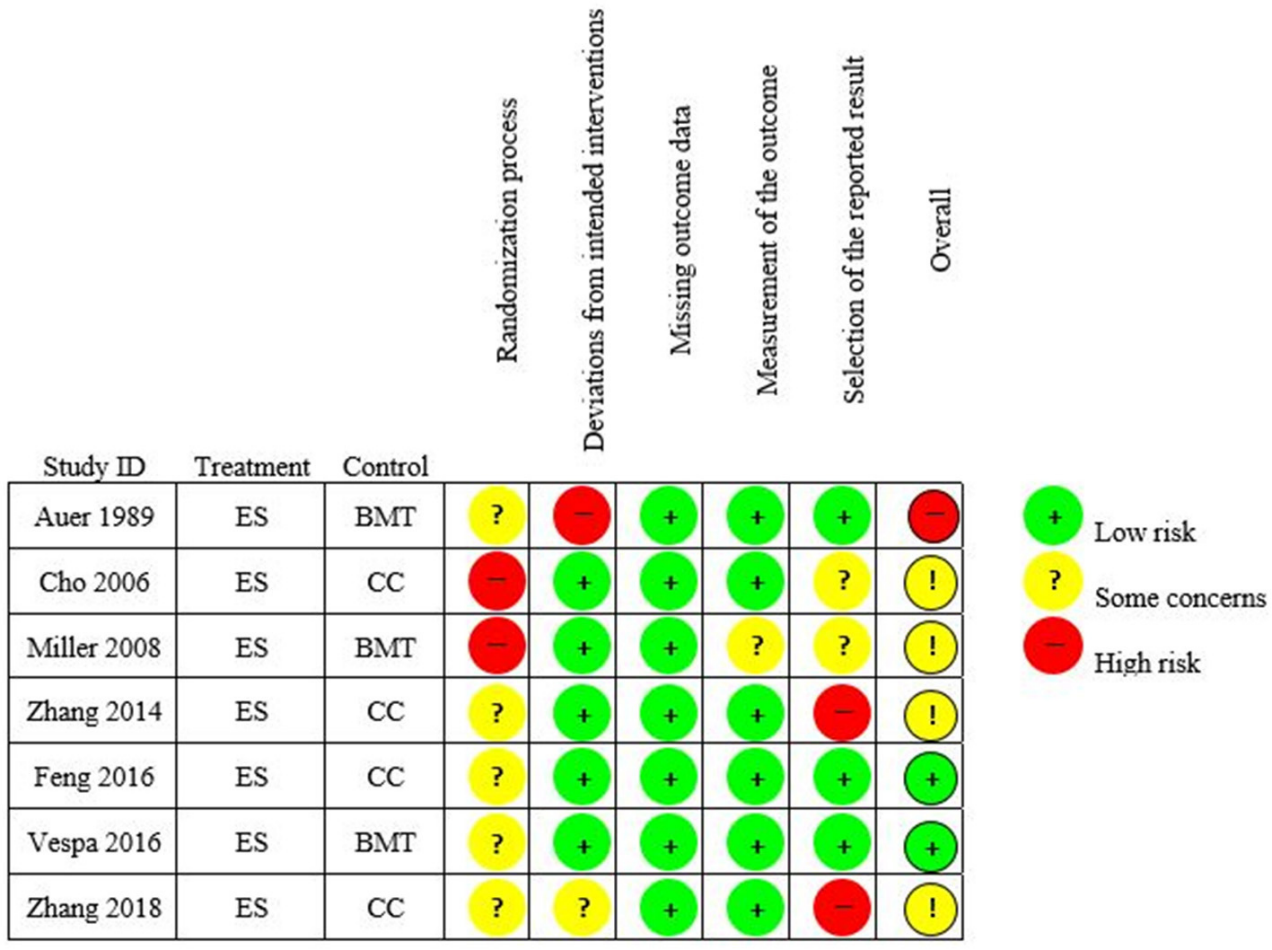
Risk of bias analysis (Rob-2) traffic light plot for the included studies.

We assessed a low overall risk of bias in two studies (35, 38), an intermediate risk of bias in four studies (34, 36, 37, 39), and a high risk of bias in one study (33). Risk of bias analysis for the included studies is shown in Figure 3.

## 4. Discussion

Our meta-analysis of the existing RCTs comparing endoscopic surgery additionally to best medical treatment to conventional craniotomy or best medical treatment alone (ES to CC or BMT) in the treatment of spontaneous supratentorial intracerebral haemorrhage (SSICH) showed that ES had a significantly higher rate of favourable functional outcome and lower 6-month mortality rate compared to BMT. Overall morbidity was significantly lower for ES compared to CC. A significantly shorter duration of surgery and non-significant smaller postoperative hematoma volume was observed for ES in contrast to CC.

Several large RCTs were conducted in the past decades investigating surgical treatment of SSICH (13–15, 17), but the role of surgery in SSICH has remained controversial (10, 16). The STICH trial included various forms of SSICH, including deep-seated and intraventricular haematomas, for which the surgeon had uncertainty concerning the choice of treatment, while STICH II only included superficial lobar haemorrhages (14, 15). However, both the STICH and STICH II trial failed to show superiority of surgical hematoma evacuation compared to BMT concerning favourable functional outcome (14, 15). In the STICH trial, 75% of all hematomas were evacuated by craniotomy and microscopic hematoma removal, while only 7% of the hematomas were evacuated endoscopically (14).

Endoscopic surgery for SSICH was first proposed by Auer et al. (33). According to their publication, the benefit of ES regarding functional recovery and mortality rates applied only to a specific subgroup of younger and healthier patients with small hematomas (33). In the past decades, several other MIS methods, including stereotactic clot aspiration, minimally invasive catheter evacuation and lysis therapy (MISTIE) or minimally invasive puncture surgery (MIPS), were compared to BMT but all failed to show a significant difference in favourable functional primary outcome analysis (11, 17). Furthermore, different meta-analyses were carried out comparing various MIS techniques to CC or BMT, resulting in different results for the respective techniques (18–25). These publications analyse mostly RCTs but also prospective and retrospective cohort studies. Further, they report ES either as main surgical technique or as a subgroup analysis but compare it to both BMT and CC combined, effectively creating a mixture of different treatments in the control group possibly limiting the generalizability of the results (18–25). To our knowledge, we present the first meta-analysis including solely RCTs of ES compared to either CC or BMT separately for the treatment of SSICH.

### 4.1. Favourable functional outcome

Our results showed a significantly higher rate of favourable functional outcome (mRS ≤ 3) for ES compared to BMT. A network meta-analysis by Guo et al. (22) comparing four interventions (BMT, ES, CC and minimally invasive puncture surgery), also observed a higher rate of favourable functional outcome in the ES group than in the BMT group. However, favourable functional outcome was defined differently in their meta-analysis with a cut off of mRS ≤ 2 (22). The observed difference in favourable functional outcome of ES and BMT might be due to a faster hematoma evacuation achieved with surgery, reducing the mass effect and disposing of neurotoxic hematoma metabolites. These were shown to negatively affect the cerebral tissue and induce brain oedema and apoptosis due to formation of reactive oxygen species (4). We assume that the evacuation of blood itself influences the outcome more than the surgical method chosen, and therefore no difference in favourable functional outcome between ES and CC was observed. However, ES is less invasive than CC, resulting in less injury of the surrounding brain tissue and shorter surgery time, which might lead to better surgical results (41).

### 4.2. Mortality and morbidity

Based on our data ES showed reduced mortality rates when compared to BMT. However, compared to CC no statistically significant difference was reached. This might indicate that hematoma evacuation, irrespective of the surgical technique, could reduce mortality by treating the mass effect, which ultimately can cause death if left untreated (4). In accordance with our results, Yao et al. described a significantly lower mortality rate for ES compared to BMT but also to CC in their meta-analysis (19). Contrary to our analysis, Yao et al. also included retrospective cohort studies and other forms of MIS and thus had a bigger sample size, which could lead to a higher heterogeneity in their data and the reported difference in outcome (19). We observed decreasing mortality rates for ES over time in the included studies (6–42%), while the mortality rates for BMT and CC remained unchanged around 43 and 8% over the time span of the different studies. We assume, that the decrease of mortality rates over time could be due to the growing experience and technological advances in ES over the years. In our analysis, we observed higher percentages of mortality in ES vs. BMT compared to ES vs. CC which could be either due to the higher number of patients included in the analysis of ES vs. CC (425) compared to ES vs. BMT (166) or due to the high mortality rate in Auer et al. (33).

Overall morbidity for ES described within the literature is rather low (8–10%) (36–38). This could be due to the improved intracavitary vision during ES. ES allows clot removal under full visual sight, and through a minimal invasive approach which could lead to the observed higher evacuation rate of ES compared to CC (42). Additionally, improved vision could not only lead to a more efficient evacuation of the clot but also improved haemostasis. In our analysis, no significant difference between ES and CC was observed concerning evacuation rates.

A common complication after SSICH are infections with reported rates of 23–38% (43, 44). We observed a significantly lower rate of any kind of infection in ES compared to CC. However, since data on the comparison of surgical site and CNS infections within the included reports is limited, most of the observed effect is attributable to reduced rates of extracranial infections. Nevertheless, ES potentially leads to less surgical site infections due to the smaller incision or the observed significantly shorter duration of surgery for ES. As proposed by Patir et al. in a prospective study, a prolonged surgery time (>4 h) significantly increases the rate of postoperative infections in neurosurgical patients (45). Hence, keeping the surgical time short appears to be of paramount importance for these patients, as severe infection could lead to worse outcome in these already frail patients.

### 4.3. Timing of the hematoma evacuation

In accordance with the well-known treatment paradigm “time is brain” in ischemic stroke, it was shown that earlier hematoma evacuation (within 24 h) could lead to a 2.8 times higher rate of functional independence compared to a prolonged evacuation period (>72 h) (18, 46). This was also suggested by a subgroup analysis of MISTIE III, which showed a trend towards better outcome in early hematoma evacuation (within 36 h) compared to later evacuation (>36 h) (17). To note, however, that ultra-early hematoma evacuation (< 4–7 h after symptom onset) was shown to be associated with a significantly higher risk of re-bleeding and a higher rate of mortality (47–49). From all the included studies only one reported ultra-early clot evacuation and no higher re-bleeding rate was observed (36). The worse outcome in ultra-early surgery could be due to diffuse bleeding and due to the poorly controlled hypertension within the 1st h after SSICH, which make a thorough haemostasis challenging. However, the literature is controversial on this topic (50). Early hematoma evacuation (after 12–24 h) is probably beneficial for achieving a good outcome in SSICH; however, further well-designed trials are needed to determine the optimal timing for surgery.

### 4.4. Limitations

Despite conducting a systematic review and meta-analysis based solely on RCTs, this study presents some limitations. First, we observed substantial heterogeneity in the analysis of several endpoints, many of which included only few studies. Given, that much of the variance of those outcomes is likely attributable to differences in the studies themselves, the absence of difference between groups must be interpreted carefully since it might not represent the absence of a true treatment difference. Second, the primary outcome (favourable functional outcome) was reported in four different outcome measures and was measured at different time points which introduces a risk of bias and limits comparison (27, 28). Third, based on the available data, we were not able to compare the outcome between lobar and non-lobar, eloquent and non-eloquent as well as deep seated and superficial seated bleedings. This limits our results when it comes to these different type of bleedings. Forth, data concerning CNS infections and SSI was sparse. Therefore, infection rates within the analysis include intra- and extracranial infections, while the true outcome of CNS infections and SSI remains elusive. Further, we only searched two of the existing databases (PubMed and Embase) and only included studies in English, which carries a risk for selection bias. The described evolution of mortality rates for ES spans over several decades with varying technical expertise and should therefore be interpreted with care. Lastly, this study is not impervious to publication bias due to possible unpublished negative results, which are not included in this meta-analysis. However, to our knowledge, this is the first meta-analysis based on RCTs solely comparing ES (and not any other forms of MIS), to CC and BMT separately.

## 5. Conclusion

To conclude, based on our pooled analysis, ES has a significantly higher rate of favourable functional outcome and lower rate of mortality compared to BMT. Further, ES showed significantly lower overall complication rate, shorter surgery time, and non-significant higher hematoma evacuation rates compared to CC. ES seems to be a promising approach in the treatment of SSICH. Nevertheless, further sufficiently powered prospective trials analysing the benefit of early ES over BMT and other surgical treatment modalities for SSICH are warranted.

## Data availability statement

The raw data supporting the conclusions of this article will be made available by the authors, without undue reservation.

## Author contributions

Conception and design: JS, LB, RG, and LG. Data curation, analysis, and interpretation of data: TH and LG. Statistical analysis and drafting the manuscript: TH, LG, and JS. Study supervision: LB, RG, and JS. Funding acquisition: LB. Critically revising the manuscript: All authors.
